# IL-34 in neurological homeostasis and its multifaceted roles in central nervous system diseases

**DOI:** 10.3389/fimmu.2026.1860233

**Published:** 2026-09-04

**Authors:** Ying Xin, Youyang Liao, Xing Cao, Xinyi Zhu, Jie Wang

**Affiliations:** Nanjing Normal University School of Life Sciences, Nanjing, China

**Keywords:** border-associated macrophages, CNS homeostasis, CSF1R, IL-34, microglia, neurodegenerative diseases

## Abstract

Interleukin-34 (IL-34), a ligand for the macrophage colony-stimulating factor receptor (CSF1R), plays an indispensable role in the development and homeostasis maintenance of the central nervous system (CNS). This review systematically outlines the core mechanisms by which the IL-34/CSF1R signaling axis regulates the development, colonization, and functional differentiation of CNS-resident immune cells-including microglia and border-associated macrophages (BAMs). It highlights its role in critical physiological processes such as synaptic pruning, neural circuit maturation, and maintenance of blood-brain barrier integrity. Beyond its physiological functions, IL-34 modulates diverse neurological disorders-such as Alzheimer’s disease, Parkinson’s disease, multiple sclerosis, ischemic stroke-with context dependent outcomes. It can confer neuroprotective effects but also drive disease progression under different conditions, providing new insights into CNS homeostasis balance and the complexity of pathological mechanisms.

## 1 Introduction

The central nervous system (CNS), as the master regulator of bodily functions, relies on a sophisticated network of immune surveillance and regulation to maintain its homeostasis. Immune cells residing in the CNS can be broadly classified into two main populations: parenchymal microglia and non-parenchymal border-associated macrophages (BAMs). As the resident immune cells of the brain parenchyma, microglia serve as key orchestrators in shaping an optimal cerebral environment (1). Their essential functions span the clearance of apoptotic cells (2, 3), including precise synaptic pruning (4–6), and the support of neuronal differentiation and survival (7, 8), ongoing immune surveillances (9), extracellular matrix maintenance (10, 11), and vascular modulation (12). Meningeal macrophages, as the most abundant BAMs, play critical roles in mediating border immune responses, modulating neuroinflammation, and contributing to disease pathogenesis (13–15). Therefore, deciphering the mechanisms that regulate the development, colonization, and functional specialization of these cells is of paramount importance, representing a major frontier in neuroimmunology research.

Interleukin-34 (IL-34), a key ligand for the macrophage colony-stimulating factor receptor (CSF1R), is a central regulator of myeloid cell fate (16–18). Within the CNS, the expression of *IL34* gene displays regional specificity, with prominent localization in critical functional areas such as the forebrain (16, 19). Through binding to its receptor CSF1R, IL-34 not only orchestrates the development, maintenance, and functional differentiation of microglia and BAMs, but also participates extensively in essential physiological processes including synaptic pruning (20), neural circuit maturation (19), and preservation of blood-brain barrier integrity (21). Research indicates that the spatiotemporal regulation of the IL-34/CSF1R signaling axis is fundamental for ensuring normal nervous system development and homeostasis.

Nevertheless, aberrant activation or dysregulation of this pathway is closely implicated in the pathogenesis of numerous neurological disorders. Current evidence suggests that the role of IL-34 is profoundly context-dependent. Based on findings from animal studies and *in vitro* experiments, IL-34 can exert neuroprotective effects by modulating microglial function, dampening inflammatory responses, and facilitating tissue repair in pathologies including Alzheimer’s disease and multiple sclerosis (22–24). Conversely, under specific pathological conditions, or within distinct microenvironments, it may also facilitate the release of inflammatory mediators, exacerbate cellular damage, or contribute to immune evasion, thereby driving disease progression. For instance, in glioblastoma (GBM), tumor cells secrete IL-34 and CSF-1, which activate the CSF1R signaling axis to induce the differentiation of monocytes into myeloid-derived suppressor cells (M-MDSCs), thereby inhibiting T-cell proliferation (25). This context-dependent multiplicity of functions establishes IL-34/CSF1R as a critical molecular node linking neural development, homeostatic maintenance, and pathology.

Our review aims to systematically summarize the role of IL-34 in CNS homeostasis and to elucidate its mechanisms of action in related diseases. We will focus on its regulatory functions concerning microglia and border immune cells, particularly meningeal macrophages, and dissect the complex network of this signaling pathway in neural development, synaptic plasticity, blood-brain barrier integrity, and disease pathogenesis. By sorting out current research advances, we endeavor to provide a theoretical foundation for a deeper understanding of neuro-immune interactions and to propose novel perspectives and directions for developing targeted therapeutic strategies for associated neurological disorders.

## 2 IL-34 and its receptors

Interleukin-34 (IL-34), a member of the interleukin-1 family, was first identified in 2008 as a ligand for the macrophage colony-stimulating factor receptor (CSF1R) (26). The human *IL34* gene is located on chromosome 16q22. In healthy tissues, IL-34 is produced by multiple cell populations such as keratinocytes, mural cells, fibroblasts, and neurons (16, 21). Under pathological conditions, its expression is markedly upregulated in fibroblasts and hepatocytes (27, 28). As a critical regulator, it plays an essential role in modulating the survival, proliferation, and differentiation of monocytes, macrophages, and myeloid dendritic cells (DCs), thereby influencing processes from development and homeostasis to disease (16, 17, 19, 20, 29). The function of IL-34 mainly depends on its interaction with the receptors on the cell membrane. IL-34 engages with a diverse array of receptors, a characteristic that substantially expands its repertoire of biological functions by activating distinct signaling pathways in different cellular contexts. It has been reported that IL-34 possesses four receptors, including CSF1R, TREM2, SDC-1, and PTP-ζ (30). Among these, CSF1R serves as its primary receptor. SDC-1 mediates the alternative binding of IL-34, regulates CSF1R activation and is required for IL-34-induced myeloid cell migration (31). In contrast, binding to PTP-ζ mediates signals that inhibit cell proliferation and migration, independent of CSF1R, closely linking IL-34’s role in regulating the maintenance and maturation of neural progenitor cells (32). Its interaction with TREM2 is implicated in myeloid malignancies such as acute myeloid leukemia (AML), offering a novel therapeutic target for these conditions (33). The functions of IL-34 within the intricate CNS are likely more extensive than solely regulating myeloid cells, given that it operates through a multi-receptor system.

## 3 Evolutionary and structural insights into IL-34 and CSF-1: a comparative analysis

In addition to IL-34, CSF-1 is another critical ligand that binds to CSF1R and share similar regulatory effects on the myeloid lineage. However, in-depth comparative analysis from evolutionary and structural perspectives reveals that IL-34 and CSF-1 are ligands of distinct origins and divergent characteristics, providing a molecular basis for their functional differences. While the differences in distribution and function between IL-34 and CSF-1 in CNS will be elaborated in the following sections, their key distinctions are summarized in **Table 1**.

**Table 1:** Comparative biological functions of IL-34 and CSF-1.

Function	IL-34	CSF-1	References
Proliferation	Primarily promotes the maintenance and local self-renewal of forebrain microglia and leptomeningeal macrophages, with a particularly critical role in gray matter regions.	Mainly drives the proliferation and survival of cerebellar microglia and developing meningeal macrophages, with a stronger effect in white matter regions.	(20, 21, 34, 35)
Migration	Provides long-range chemotactic signals in zebrafish models, guiding macrophage precursors to colonize regions such as the forebrain and retina.	More involved in the early colonization of perivascular macrophages, closely associated with developmental angiogenesis.	(17, 21)
Polarization	Tends to promote microglial/macrophage state shift, characterized by increased IL-10 and TGF-β expression and supporting vascular homeostasis and post-ischemic repair.	More supportive of an inflammatory macrophage state, associated with the survival of inflammatory macrophages and greater pathogenicity in autoimmune models.	(36–38)
Major Target Cells	leptomeningeal macrophages, forebrain microglia, and some perivascular macrophages.	Cerebellar microglia, microglia in white matter, developing meningeal macrophages and monocyte/macrophage lineages	(20, 21, 34)
Disease Association	Protective: • Early AD (hippocampus): Promotes Aβ clearance, upregulates IDE/HO-1, suppresses NLRP3 inflammasome via TREM2, and ameliorates cognitive impairment in APP/PS1 mice. • Aging/EAE (cortex): Expands an autophagy-dependent microglial subpopulation (ADAM), protecting against neuronal and glial death. Pathological: •Late AD (cortex, 3x-Tg mice): Chronic IL-34 exacerbates anxiety, memory loss, amyloidosis, tau phosphorylation, and RAGE expression. •HD (striatum, brain slice): IL-34/CSF1R signaling accelerates mHTT-induced medium spiny neurons (MSNs) degeneration. •Prion disease (cortex, hippocampus, thalamus): IL-34 shifts from neurons to reactive glia, potentially promoting neuroinflammation.	Pathological •PD (substantia nigra): CSF-1 is significantly upregulated, correlating with microgliosis and dopaminergic neuron loss. • HIV-1: CSF-1-polarized macrophages show lower viral resistance than IL-34-polarized cells.	(23, 39–44)
Receptor Diversity	Can bind to CSF1R, TREM2, PTP-ζ, SDC-1, resulting in more pleiotropic and microenvironment-dependent signaling.	Primarily binds to CSF1R, with functions more specialized in myeloid cell survival and differentiation.	(30–32, 36)
Evolutionary Conservation	Relatively high, conserved across vertebrates, suggesting an ancient role in fundamental immune-neural regulation.	Relatively lower, functions more focused on myeloid development and bone metabolism in mammals.	(45)

**Figure 1:**
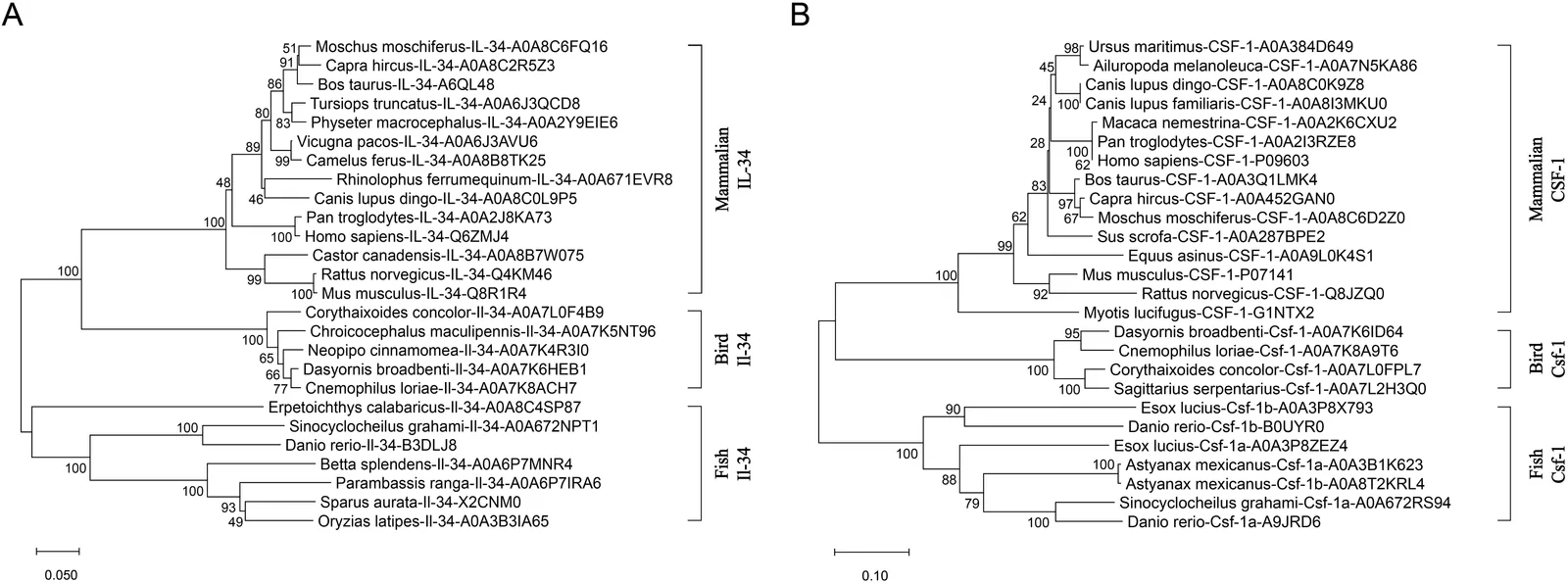
**(A)** An unrooted phylogenetic tree illustrating relationships among partial fish Il-34 molecules, partial bird Il-34 molecules, and partial mammalian IL-34 molecules. **(B)** An unrooted phylogenetic tree for partial fish Csf-1 molecules, partial bird Csf-1 molecules, and partial mammalian CSF-1 molecules. Both trees were constructed using the neighbor-joining method in the MEGA5 program based on amino acid multiple sequence alignments, with the p-distance model employed to directly calculate the proportion of amino acid differences between sequences. Bootstrap tests were conducted with 500 replicates. The accession number for each sequence is provided adjacent to it.

Constructing phylogenetic trees of the functionally related ligand IL-34 and CSF-1 across diverse species, such as humans and mice, provides critical insight into their distinct evolutionary trajectories (**Figure 1**). This analysis reveals that while both proteins ultimately serve as ligands for the same receptor, CSF1R, their origins and evolutionary histories are remarkably different. The CSF-1 is present in a wide range of jawed vertebrates (gnathostomes). However, CSF-1 is notably absent from cyclostomes (jawless fishes, e.g., lampreys), and orthologs are either incompletely recovered or absent in cartilaginous fishes (e.g., sharks). This pattern suggests that CSF-1 emerged following the divergence of these early vertebrate lineages (45). The evolutionary history of CSF-1 is further marked by lineage-specific duplication events. For instance, in teleost fish, *csf1* gene has undergone duplication, giving rise to two paralogous genes, “*csf1-a*”and”*csf1-b*” (46). This duplication likely allowed for subfunctionalization or neofunctionalization, contributing to the diversification of immune cell populations in this lineage. In contrast to CSF-1, IL-34 exhibits a broader and more ancient distribution, pointing to its emergence much earlier in vertebrate evolution. Phylogenetic analysis detects IL-34 in the coelacanth, a representative of early jawed fishes, and even in cartilaginous fishes like the whale shark. Its presence in these basal lineages indicates that IL-34 was present in the common ancestor of all jawed vertebrates (45). The deep conservation of IL-34 orthologs is further underscored by its clear species-specific clustering in phylogenetic trees. Mammals (including whales and bats), birds, and fish each form independent, highly supported monophyletic groups. This evolutionary path aligns closely with established taxonomic classifications, spanning a broad phylogenetic range and demonstrating the widespread presence and purifying selection acting on the *IL34* gene across vertebrates. This has led to the hypothesis that *IL34* has a more ancient evolutionary origin than previously recognized (45). This conclusion is consistent with the phylogenetic tree we constructed, which does not include jawless vertebrates, while the basal jawed fish branches support the ancient origin of *IL34* gene. In contrast, syntenic evidence indicates that *CSF1* emerged later, inserting into conserved genomic loci after the divergence of jawed fishes. Although IL-34 and CSF-1 exhibit significant divergence in amino acid sequence and have distinct evolutionary origins, their subsequent parallel co-evolution with CSF1R suggests that they have undergone convergent evolution to preserve conserved functions for the same receptor.

From the perspective of protein structure and functional characteristics, although IL-34 and CSF-1 exhibit low sequence homology at both DNA (~26%) and protein levels, they share a canonical four-helix bundle cytokine fold core. Their main structural differences are as follows: Firstly, IL-34 possesses a longer amino acid sequence (eg. IL-34: 242 AA vs. CSF-1: 192 AA) with unique N- and C-terminal extensions, and it replaces two β-strands with two short helices (36, 47, 48). Notably, the C-terminal region of IL-34 is heavily glycosylated and adopts a highly disordered conformation, a feature that may influence its stability and regulatory role in signaling (49). In terms of receptor-binding mechanisms, both cytokines interact primarily with similar sites on the immunoglobulin-like domains D2 and D3 of the CSF1R extracellular region. However, the nature of their binding differs significantly: IL-34 predominantly relies on hydrophobic interactions, whereas CSF-1 is driven more by hydrophilic interactions (48). This structural distinction is directly reflected in their binding affinities: IL-34 exhibits a significantly higher affinity for CSF1R (Kd ≈ 1 pM), far exceeding that of CSF-1 (Kd ≈ 34 pM) (26). Such a difference in affinity may further contribute to their distinct signaling activation kinetics and functional specificities.

## 4 Introduction of the IL-34/CSF-1-CSF1R signaling pathway

Our review focuses on the signaling pathway mediated by IL-34/CSF-1 and its macrophage colony-stimulating factor 1 receptor (CSF1R). This is because the survival, proliferation, differentiation, and recruitment of almost all mononuclear phagocytes depend on this pathway (16, 50–52). Specifically, the ligands of CSF1R, CSF-1 or IL-34, bind to and activate the tyrosine kinase receptor CSF1R located on mononuclear phagocytes. This activation triggers a cascade of intracellular pathways, thereby regulating the development and maintenance of phenotypic characteristics in these cells. Within the CNS, the CSF-1/IL-34-CSF1R signaling pathway plays a crucial role in the establishment and maintenance of microglia in the brain parenchyma (16, 17, 34). In humans, mutations in the *CSF1R* gene lead to severe microglial depletion and neurodegenerative pathologies (53, 54). Studies using murine models have revealed distinct expression patterns and functional roles for the two known ligands of CSF1R, CSF-1 and IL-34, within the brain parenchyma. CSF-1 is expressed predominantly by the astrocyte lineage within the cerebellum, with additional expression observed in mature neurons of both the cerebellum and dorsal forebrain, and it is essential for regulating microglial development in the cerebellum and white matter (34, 35). In contrast, IL-34, predominantly responsible for the development and maintenance of microglia in gray matter regions and perivascular macrophages, is mainly expressed by neurons, mural cells, and fibroblasts (20, 21, 35). Compared to CSF-1, IL-34 exhibits a higher binding affinity for CSF1R, which aligns with its role in sustaining microglial homeostasis in its specific functional domains (49). Furthermore, this spatial and functional partitioning is not strictly independent but rather constitutes a complementary and synergistic regulatory network. For instance, in the hippocampus of adult mice, simultaneous blockade of both ligands results in significantly more effective microglial depletion compared to individual inhibition of either ligand alone (35). This indicates that when signaling from one ligand is disrupted, the other can provide partial compensatory support to sustain microglial populations. Additionally, under pathological conditions such as neurodegenerative diseases, neuroinflammation, or brain injury, the expression profile and function of IL-34 may undergo dynamic reprogramming, exhibiting reaction patterns distinct from those of CSF-1, suggesting that they play differentiated roles in disease progression (55, 56). It remains to be further explored what upstream regulatory signals mediate its differences from CSF-1.

## 5 IL-34/CSF1R signaling pathway in the development of the central nervous system

### 5.1 Functions of IL-34 action on BAMs development

BAMs are a population of resident macrophages located at the interfaces between the CNS and the external environment or the bloodstream, including those in the perivascular spaces, choroid plexus, and meninges (dura mater, leptomeninges) (57, 58). Acting as immune sentinels at CNS borders, they play crucial roles in surveillance, defense, and the maintenance of brain homeostasis.

The traditional view held that meningeal macrophages originated from the bone marrow. However, recent lineage tracing studies indicate that majority of resident macrophages in the CNS, including both microglia and BAMs, originate from the embryonic yolk sac, not from adult bone marrow-derived monocytes (59–62). These yolk sac-derived macrophage precursors begin colonizing the CNS during early embryonic development (e.g., around E9.5 in mice) and subsequently disseminate to various border regions (59, 63). Further research has revealed that there exists transcriptional heterogeneity within the erythro-myeloid progenitors (EMP) population, and their fate depends on the expression of the BAM-specific marker CD206 and the timing of differentiation. CD206^+^ cells can differentiate into either microglia or BAMs, and CD206^-^ macrophages infiltrating from the ventricles can even transform into microglia (62, 64, 65). Subsequently, BAMs exhibit significant heterogeneity and tissue specificity. Based on anatomical location, meningeal macrophages can be divided into dura mater macrophages and leptomeningeal macrophages. Further analysis via single-cell RNA sequencing and surface marker profiling reveals that dura mater macrophages can be subdivided into MHC II high-expressing (Dhi-BAM) and low-expressing (Dlo-BAM) subsets, each enriched for distinct immune function-related pathways (13, 14). The observed heterogeneity indicates functional diversification of BAMs across varied microenvironments. Subsequent investigations should characterize the molecular basis underlying their functional divergence, illustrate how microenvironmental cues modulate BAM function, and assess the involvement of epigenetic regulation in preserving their heterogeneous features.

**Figure 2:**
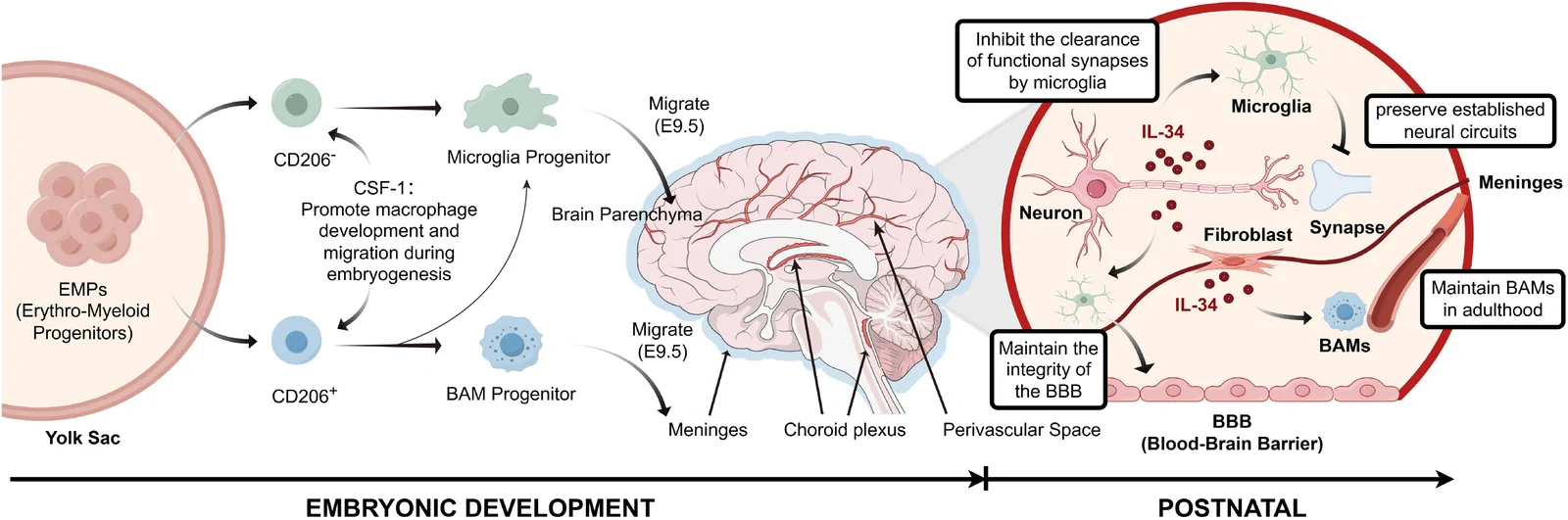
Development, colonization, and function of CNS immune cells. (Left Panel) Embryonic Development: Both microglia and border-associated macrophages (BAMs) originate from erythromyeloid progenitors (EMPs) in the yolk sac. During lineage specification, CD206- primitive macrophages give rise to microglial progenitors, whereas the majority of CD206+ cells give rise to BAMs, while a small subset enters the pallium to become microglia. Around embryonic day 9.5 (E9.5), these progenitor cells migrate and colonize the central nervous system (CNS), with microglial precursors entering the brain parenchyma and BAM precursors settling in the meninges and perivascular spaces. Throughout prenatal development, CSF-1 acts as the primary driving factor promoting embryonic macrophage proliferation, development, and early niche colonization. (Right Panel) Postnatal Maturation and Functional Homeostasis: During the postnatal and adult stages, the long-term maintenance and physiological functions of CNS macrophages rely on compartment-specific IL-34 signaling. Neuron-derived IL-34 sustains postnatal microglial survival and proliferation. Concurrently, neuronal IL-34 suppresses excessive synaptic pruning by microglia, thereby preserving functional neural circuits, while also maintaining blood-brain barrier (BBB) integrity through the upregulation of tight junctions (TJs). Fibroblast-derived IL-34 serves as the crucial niche factor maintaining BAM survival and population density within border compartments. Parenchymal microglia and border-associated BAMs (in coordination with perivascular fibroblasts) synergistically maintain the structural and functional integrity of the blood-brain barrier (BBB). Created with Figdraw.

The colonization, differentiation, and maintenance of BAMs strictly depends on signaling input through their receptor CSF1R, but its ligands CSF-1 and IL-34 play dominant roles at different stages of the BAM lifecycle and in different spatial compartments. Studies indicated that during mouse brain development, the initial differentiation and colonization of BAMs, including those in the choroid plexus, leptomeninges, and perivascular spaces, are primarily driven by CSF-1. The significant reduction in the number of embryonic BAMs in the absence of CSF-1 indicates that CSF-1 is a key regulator of their early development (21). However, upon entering adulthood, the mechanism for long-term homeostasis of BAMs switches to being predominantly mediated by IL-34. Research shows that although IL-34 is dispensable during the embryonic period, in the adult brain, IL-34 derived from mural cells (such as pericytes and vascular smooth muscle cells) and fibroblasts is a core signal for maintaining BAM survival (21). Global knockout of *Il34* markedly reduces macrophage numbers in the choroid plexus, leptomeninges, and perivascular spaces of adult mice, and conditional ablation of *Il34* in mural cells and fibroblasts using *Tbx18*^CreER^;*Il34*^fl/fl^ mice recapitulates the same phenotype (21). This “ligand switch” from CSF-1 to IL-34 exemplifies the precise temporal regulation of this signaling pathway (**Figure 2**).

IL-34 signaling has important functional consequences for BAM maintenance and function. For instance, *Il34* global knockout mice show reduced numbers of perivascular macrophages accompanied by hemodynamic abnormalities, suggesting that BAMs interact functionally with the cerebral vasculature through an IL-34-dependent mechanism, which is crucial for maintaining cerebrovascular homeostasis (21). Furthermore, as the first line of immune defense at CNS borders, BAMs (particularly meningeal macrophages) play significant roles in pathological processes. For example, they can restrict viral infection, prevent fatal meningitis, and present cerebrospinal fluid -borne antigens to patrolling T cells for immunosurveillance (14, 15, 66), and show altered activity in diseases such as leptomeningeal metastasis (67). Thus, modulating the number, phenotype, or function of BAMs by targeting the IL-34/CSF1R signaling pathway offers a promising new approach for treating cerebrovascular diseases, neuroinflammation, infections, and neoplastic meningeal disorders. Future research directions include investigating recombinant IL-34 protein interventions, refining routes of administration, and advancing the development and application of IL-34-specific agonists or inhibitors.

Notably, IL-34 exhibits a restricted expression pattern in peripheral tissues, having been detected in keratinocytes, placental cyto- and syncytio-trophoblasts, decidual stromal cells, hair follicles, proximal kidney, germinal cells (68), as well as in CD4^+^FOXP3^+^ and CD8^+^FOXP3^+^ regulatory T cells (69). Conversely, CSF-1 is more broadly and constitutively expressed by fibroblasts, endothelial cells, stromal cells, monocytes/macrophages, osteoblasts, as well as by various malignant cells such as AML blasts and lymphoma cells (70). Consequently, the circulating concentration of IL-34 in the periphery is very low (approximately 52 pg/mL) (71), while the peripheral circulating concentration of CSF-1 is relatively higher than that of IL-34, at approximately 4.5 ng/mL (70). CSF-1 is capable of penetrating an intact blood–brain barrier (72). Findings from animal studies suggested that peripheral administration of CSF-1 can promote the entry of myeloid cells into the brain and their transformation into microglia-like cells, thereby clearing β-amyloid (Aβ) plaques and ameliorating or preventing cognitive decline in mouse models of Alzheimer’s disease (73). Therefore, CSF-1 may act as a regulatory signal between peripheral and central myeloid compartments, while IL-34 functions as a locally restricted signal. These observations are consistent with the notion that local secreted IL-34 can maintains BAM homeostasis and microglia in the brain through paracrine signaling via CSF1R and peripheral IL-34 blockade has been shown not to reduce microglial numbers (21, 74). The functional disparities and regulatory mechanisms between locally produced IL-34 and peripherally derived CSF-1 in maintaining BAM homeostasis also require in-depth investigation.

### 5.2 Roles of IL-34 action on microglial development

Following their migration into the CNS during early embryogenesis, the survival, proliferation, and differentiation of microglia are critically dependent on CSF1R signaling (19, 53). The receptor CSF1R deficiency leads to the near-complete depletion of microglia, with the remaining cells being functionally impaired and unable to effectively support neuronal development and homeostasis (53), a phenotype that mirrors that of deficiency both of CSF-1 and IL-34 (35). In addition to synergizing with CSF-1, IL-34 is an essential regulator of mouse microglia, with its activities tightly linked to developmental stages. During embryonic development, antibody blockade experiments have indicated that IL-34 signaling is dispensable for the initial colonization of microglial progenitors in mice: peripheral administration of anti-IL-34 antibodies at E6.5 and E7.5 (assessed at P0.5) did not significantly affect microglial numbers in any brain region examined. In contrast, CSF-1 blockade during the same period led to a marked reduction in microglial density across all brain regions, indicating that CSF-1, but not IL-34, is the principal ligand governing microglial seeding during embryogenesis (35). The role of IL-34 becomes evident in the early postnatal period in mice. Administration of anti-IL-34 antibodies from P0.5 to P4 resulted in a significant decrease in microglial density in the gray matter, confirming that IL-34 begins to contribute to microglial maintenance from this stage onward (35). This effect persists into adulthood, as evidenced by the regional reduction of microglia observed in adult *Il34* ^LacZ/LacZ^ mice, further supporting that IL-34 is essential for the maintenance of microglial homeostasis during adulthood (75). Notably, using zebrafish as an animal model, one study revealed that in the context of IL-34 systemic deficiency, embryonic macrophages develop normally in peripheral tissues but rarely enter the CNS (17). These findings indicate that the IL-34/CSF1R signaling axis functions in a species-specific manner: in some species (e.g. zebrafish), it acts as a long-range chemotactic signal guiding microglial precursors into the CNS; in some other species (e.g. mice), it instead provides essential survival cues for sustaining resident microglia within the brain. However, the molecular mechanisms underlying such species-specific functions remain poorly understood. Further studies are needed to compare the regulatory networks of this pathway among various species, and to explore whether the divergent functions are driven by sequence variation of IL-34/CSF-1, differences in expression patterns, or distinct downstream signaling cascades.

IL-34 also demonstrates profound region-specificity in its effects. Functional blocking experiments revealed that administration of a specific neutralizing antibody against IL-34 in adult mice significantly depleted microglia in gray matter regions, such as the cerebral cortex and striatum, achieving a depletion rate of 40-50% as quantified by immunofluorescence combined with two-photon microscopy. However, microglia in white matter tracts, including the hippocampal fimbria and corpus callosum, remained largely unaffected (35). In transgenic mouse models, this regional selectivity is even more specific. Using an *Il34* reporter mouse strain (*Il34*^LacZ/+^) combined with X-gal staining, IL-34 expression in the adult brain was found to be highly restricted to specific regions, such as the cortex, hippocampus, and anterior olfactory nucleus, with minimal to no expression detected in the brainstem and cerebellum (75). Consequently, in adult *Il34*-deficient mice (*Il34*^LacZ/LacZ^), microglial numbers were reduced by approximately 50% specifically in these IL-34-high regions (e.g., cortical gray matter), while remaining largely unchanged in IL-34-low regions (75). This local maintenance defect further substantiates IL-34’s role as a “region-selective maintainer” within the CNS and explains why the absence of IL-34 signaling leads to a significant reduction of microglia in specific brain areas. In contrast, CSF-1 serves as the primary maintenance factor for the microglia resident in the white matter and cerebellar microglia (34, 35). Collectively, these findings indicate that CSF-1 and IL-34 play regionally specialized roles in the development and maintenance of microglia. These researches indicate that the CSF-1/IL-34-CSF1R signaling axis provides both long-distance chemotactic cues, which guide microglial precursor cells to populate the CNS, and essential survival signals for the maintenance of resident microglia within the brain.

Remarkably the function of IL-34 extends throughout the lifespan. In the aging stage, the role of IL-34 expands further, as it has been suggested to promote the expansion of a newly identified, autophagy-dependent microglial subpopulation (ADAM), thereby exerting a protective function during neuroinflammation (24). In the context of neurodegenerative disorders such as Alzheimer’s disease (AD), IL-34 exerts neuroprotective functions by directly modulating microglia. Using the SAMP8 mouse model of sporadic AD, Wang et al. demonstrated that continuous intracerebroventricular infusion of IL-34 for four weeks into aged AD-model mice improved cognitive function, as confirmed by the Morris water maze test (39). In summary, IL-34 not only intricately regulates the quantity and distribution of microglia through developmental stage-specific and region-specific actions but also plays multifaceted roles throughout development, maturity, and aging, transitioning from a regulator of functional maturation to a key neuroprotective factor.

### 5.3 Synaptic pruning and neural circuit development

The IL-34/CSF1R signaling pathway plays a crucial role in synaptic pruning and neural circuit maturation. Devlin et al. (2025) revealed that IL-34 expression is significantly upregulated in excitatory neurons (VGLUT2+) during the second postnatal week in mice, driving the transition of microglia from a phagocytic state to a mature homeostatic phenotype (TMEM119+) via CSF1R signaling, thereby acting as a signal that inhibits excessive synaptic pruning. With global or excitatory neuron-specific *Il34* knockout, microglia in the anterior cingulate cortex and hippocampal CA1 region remained in an immature state, characterized by reduced homeostatic markers (TMEM119, P2RY12) and enhanced lysosomal activity (CD68), and aberrantly reactivated phagocytic function after the critical postnatal 15 period, excessively engulfing VGLUT2+ presynaptic structures and leading to a significant reduction in functional synapses (VGLUT2+/PSD95+). This subsequently resulted in reduced ultrasonic vocalizations during early development, anxiety-like behavior in adulthood, and cognitive flexibility deficits (20). This suggests IL-34 signaling suppresses microglial clearance of functional synapses, thereby protecting established neural circuits. This also provides novel therapeutic targets for neurodevelopmental disorders. In the hippocampus, erythropoietin-induced neuronal differentiation is accompanied by IL-34 downregulation, thereby suppressing microglial proliferation and activity. This IL-34 downregulation mechanism provides a developmental environment for newborn neurons by reducing microglial numbers and activity (76). In summary, the IL-34/CSF1R signaling axis maintains the homeostasis of neural circuits by dynamically modulating microglial activity during neurogenesis to support the integration and maturation of newborn neurons regulating microglia-mediated synaptic pruning. Consequently, IL-34/CSF1R signaling plays a crucial role in neural development and plasticity.

### 5.4 Role of the IL-34/CSF1R signaling pathway in blood-brain barrier

The blood-brain barrier (BBB) is formed by microvascular endothelial cells lining the inner walls of cerebral capillaries. It maintains central nervous system homeostasis by restricting the passage of plasma components, red blood cells, and immune cells from the circulating bloodstream, playing a crucial role in protecting brain parenchyma from bloodborne pathogens (77) Tight junction (TJ) proteins are essential for the functional integrity of the blood-brain barrier. Studies indicate that mouse brain capillary endothelial cell lines MBEC4 cells highly express CSF1R. When pro-inflammatory cytokines (such as IL-1β and TNF-α) inducing BBB disruption, IL-34 restores the BBB by upregulating TJ expression levels (e.g. claudin-5 and occludin), suggesting neurons regulate BBB function via IL-34 (78). Additionally, IL-34 deficiency in mural cells and perivascular fibroblasts triggers vascular cell transcriptional reprogramming through the IL-34/CSF1R-BAMs pathway, leading to enhanced flow velocity and vasomotion in pial and penetrating arterioles (21). Concurrently, CSF1R mutations impair the receptor’s ability to respond to its ligands, CSF-1 or IL-34, which attenuates downstream ERK signaling. Consequently, this signaling defect drives BBB disruption and concurrently reduces the phagocytic capacity of BAMs. In contrast, microglial phagocytosis remains largely unaffected, which could be attributed to the development of compensatory mechanisms, or to an intrinsically lower signaling threshold for CSF1R in microglia (79). In addition, recent research provides direct evidence supporting the central role of this pathway in regulating BBB from an upstream regulatory perspective. ADAMTS18 is a metalloproteinase involved in extracellular matrix regulation. Studies using *Adamts18* knockout mouse models have shown that ADAMTS18 deficiency leads to structural impairment and compromised integrity of the blood-brain barrier, accompanied by significant downregulation of key signaling molecules in the brain, including IL-34 and CSF1R (80). This finding establishes ADAMTS18 as an important upstream regulator of the IL-34/CSF1R pathway and further demonstrates that dysregulation of ADAMTS18/IL-34/CSF1R pathway is a key mechanism underlying blood-brain barrier defects (80). Together, these findings demonstrate that the IL-34/CSF1R signaling pathway is essential for maintaining BBB homeostasis and raise the possibility that upregulating IL-34 within the central nervous system could serve as a novel therapeutic strategy against neuroinflammation and neurodegenerative disease. However, key questions are yet to be addressed: How exactly does IL-34 modulate BBB-associated cells (endothelial cells, pericytes, astrocytes, border-associated macrophages)? Does it act solely through CSF1R, and how do its effects compare with or differ from those of CSF-1? What are the optimal dosage, delivery route, and safety profile for CNS IL-34 upregulation? Does its effect on BBB integrity depend on disease stage, and can it synergize with other BBB-stabilizing pathways? Answering these questions is critical for translating IL-34-based therapies into clinical practice.

## 6 Abnormal IL-34 signaling in neurological diseases and its related potential therapeutic strategies

### 6.1 Alzheimer’s disease

Alzheimer’s disease (AD), the most prevalent neurodegenerative disorder, is characterized by key pathological features including abnormal accumulation of amyloid-(Aβ) into amyloid plaques, hyperphosphorylation of tau protein leading to neurofibrillary tangles and persistent neuroinflammation (81, 82). These pathological changes collectively contribute to neuronal structural and functional damage, as well as synaptic loss, ultimately resulting in memory impairment and executive dysfunction. The pathogenesis of AD primarily involves aberrant immune responses mediated by microglia and macrophages. Notably, in a preclinical Alzheimer’s disease animal model, elevated inflammatory mediators in the brain are associated with the progression of AD-like pathology (83).

IL-34 exerts stage-dependent effects in AD. In the early stages of disease in a sporadic AD mouse model, intracerebral IL-34 levels progressively decrease. Mechanistically, in primary microglia stimulated with Aβ42, IL-34 pretreatment suppressed NLRP3 inflammasome activation and proinflammatory cytokine release such as IL-1β and TNF-α; lentivirus-mediated TREM2 knockdown and co-immunoprecipitation assays confirmed that IL-34 directly binds to TREM2, and that TREM2 knockdown abolished its anti-inflammatory effects. Based on these findings, it is suggested that supplementation of exogenous IL-34 may inhibit NLRP3 inflammasome activation in microglia and reduce proinflammatory cytokine release via the TREM2 signaling pathway, thereby alleviating neuroinflammation and oxidative stress damage (39). Experimental studies demonstrate that continuous ventricular infusion of IL-34 improves spatial cognitive deficits in SAMP8 model animals and restores neuronal and synaptic marker expression. Additionally, *in vitro* experiments confirm that IL-34-activated microglia, by inducing TGF-β production, exhibit suppressed proliferation and enhanced neuroprotective functions (40). Furthermore, in the hemi-forebrains of APP/PS1 transgenic mouse model of AD, IL-34 has been reported to reduce oligomeric Amyloid-β (oAβ) levels and ameliorate cognitive impairment through upregulating Insulin-Degrading Enzyme (IDE) and Heme Oxygenase-1 (HO-1), supporting the notion that IL-34 enhances the neuroprotective function of microglia against oAβ neurotoxicity (23). However, in late-stage AD or specific models (e.g., 3xTg AD mice), IL-34 exacerbates pathological features. Studies show that sustained IL-34 administration increases the release of pro-inflammatory cytokines and aggravates anxiety-like behavior, memory impairment, amyloid deposition, tau phosphorylation, and RAGE expression in cortex (41). In summary, IL-34 plays a context-dependent, stage-specific role in AD, exhibiting neuroprotective and anti-inflammatory effects in early disease phases while potentially contributing to pathological progression in advanced stages.

Regarding AD treatment, in SH-SY5Y human neuroblastoma cell line vitamin D (1,25-dihydroxyvitamin D3) can directly induce IL-34 expression via the vitamin D receptor (VDR) in SH-SY5Y human neuroblastoma cell line, which raises the possibility that vitamin D signaling may modulate IL-34 levels in the context of AD (84). Additionally, in a mouse model of sporadic Alzheimer’ disease, selectively administration of IL-34 during the phase of significant neuroinflammation can reduce microglial proliferation without affecting peripheral macrophages and mitigate oxidative stress, thereby improving cognitive function (39). Furthermore, targeting the TREM2 receptor has emerged as a highly promising research direction. Studies reveal that TREM2 molecule possesses a specific structural domain for IL-34 binding, providing a theoretical basis for developing small-molecule drugs that precisely modulate microglial function (22, 85).

Given the dual stage-dependent roles of IL-34 in Alzheimer’s disease (AD), multiple intriguing research avenues remain to be explored. It is critical to clarify the intrinsic mechanisms driving the functional switch of IL-34 from neuroprotection to disease exacerbation during AD progression, and to define the exact time window for safe and effective IL-34 intervention. Moreover, developing selective agents targeting the IL-34/TREM2 interaction holds great translational potential; future studies could focus on designing targeted small molecules to precisely regulate microglial activity, avoid adverse impacts on peripheral immune cells, and block the detrimental effects of IL-34 in late AD. In addition, combinatorial strategies integrating timed IL-34 supplementation, vitamin D intervention and TREM2-targeted therapy deserve in-depth investigation to achieve better therapeutic outcomes for AD.

### 6.2 Parkinson’s disease

Parkinson’s disease (PD) is a common neurodegenerative disorder characterized by motor symptoms including resting tremor, muscle rigidity, and bradykinesia, accompanied by non-motor symptoms such as cognitive declines and sleep disturbances (86). The pathological mechanism of PD involves progressive loss of dopaminergic neurons in the substantia nigra of the midbrain and abnormal aggregation of α-synuclein to form Lewy bodies (87). Growing evidence indicates that altered expression of specific inflammatory protein markers such as IL-6, IL-4, and IFN-γ in the peripheral blood of PD patients (88). Moreover, microglia exposed to low-dose lipopolysaccharide leads to the degeneration of dopaminergic neurons (89), underscoring the critical role of neuroinflammation in the pathogenesis of PD.

The CSF1R signaling pathway plays a critical role in the regulatory network of neuroinflammation in Parkinson’s disease (PD), with its two primary ligands, CSF-1 and IL-34, exhibiting distinct expression patterns in PD (42). Notably, the regulation of IL-34 is more “context-dependent”: unlike CSF-1, which is consistently upregulated in response to both dopaminergic neuron injury (MPTP model) and inflammatory stimulation (LPS model), IL-34 is significantly elevated only under potent inflammatory conditions (e.g., repeated LPS injections) and remains unchanged in the neuron injury-dominant MPTP model (42), their actual modes of participation in PD pathology differ substantially. In PD patients, this divergence also manifests as a systemic imbalance in CSF1R signaling: on one hand, CSF-1 expression is significantly upregulated in the substantia nigra of PD patients, whereas IL-34 expression shows no significant change in this brain region. However, given that Parkinson’s disease involves multiple brain regions beyond the substantia nigra, such as the olfactory bulb, temporal lobe, and brainstem nuclei—which are implicated in non-motor symptoms and early Braak stage pathology (90), examining *IL34* expression in these distinct brain regions may provide further insight into its region-specific roles in PD pathogenesis.

Pharmacological interventions targeting CSF1R hold promising therapeutic potential. Studies have demonstrated that the use of selective CSF1R inhibitors (e.g., GW2580 and PLX3397) effectively suppresses microglial proliferation and pro-inflammatory activation in the substantia nigra and striatum driven by both CSF-1 and IL-34, thereby achieving transient depletion of aberrantly activated microglia and modulating the release of inflammatory mediators (42, 55, 91). This, in turn, improves behavioral outcomes and enhances dopaminergic neuronal survival in animal models of PD. Furthermore, matrix metalloproteinase-8 (MMP8) inhibitors have also shown protective effects in PD-associated neuroinflammation. In aged normal mice and LRRK2 G2019S Parkinson’s disease model mice, M8I treatment inhibited glial cell activation and the expression of inflammatory factors induced by LPS, and improved motor functions. Furthermore, *in vitro* experiments confirmed that M8I suppressed microglial inflammatory responses potentiated by CSF-1 or IL-34, reducing the production of TNF-α and NO (92). These findings suggest that MMP-8 may function as a downstream effector molecule of the CSF-1/IL-34-CSF1R signaling axis, jointly participating in the regulation of neuroinflammatory responses in PD. Together, targeting the CSF1R signaling pathway represents a novel therapeutic strategy for modulating PD-related neuroinflammation. Currently, most studies on CSF1R signaling in PD typically employ tools that block both CSF-1 and IL-34 simultaneously (e.g., antibodies or inhibitors), or focus exclusively on CSF1R itself. Studies specifically designed to distinguish the unique functions of these two ligands remain limited, largely due to their potential functional redundancy, which poses substantial experimental challenges. Hence, more sophisticated and innovative approaches are required to differentiate their biological roles in future research.

### 6.3 Huntington’s disease

Huntington’s disease (HD) is an autosomal dominant neurodegenerative disorder caused by abnormal expansion of the CAG trinucleotide repeat in the first exon of the HTT gene (93), characterized by selective loss of neurons in the striatum and cortex (94). The mutation leads to the production of a toxic mutant huntingtin protein (mHTT) containing an expanded polyglutamine (polyQ) tract (95). mHTT is readily cleaved by proteases into mutant huntingtin exon 1 fragments (mHTTx1) with amyloid-like properties. These fragments aggregate within neurons and induce neurotoxicity, including abnormal signaling pathway activation, altered gene expression, and neuronal death, which ultimately leads to progressive motor dysfunction (e.g., choreiform movements), cognitive decline, and psychiatric symptoms (95, 96).

Studies indicates that the aggregation of mHTTx1 in neurons activates Inhibitor of nuclear factor kappa-B kinase subunit β(IKKβ), which in turn induces the expression and secretion of IL-34. However, a direct regulatory relationship between IKKβand the IL-34/CSF1R axis has not been conclusively established. Experimental evidence shows that mHTTx1 significantly upregulates both *Il34* mRNA and IL-34 protein levels in human embryonic stem cell-derived dopaminergic neurons, whereas wild-type HTT exhibits no such effect (43).

IKKβ plays a core regulatory role in this process: either shRNA-mediated knockdown of IKKβ or its pharmacological inhibition using small molecules such as luteolin, TPCA, or sodium salicylate completely blocks mHTTx1 aggregation and IL-34 production, and also reduces levels of the apoptosis-associated protein procaspase-3 (43). Furthermore, in brain slice models with intact neuron-glia networks, IL-34 acts on microglia by binding its receptor CSF1R, exacerbating mHTTx1-induced degeneration of medium spiny neurons (MSNs) in the striatum (43). As a natural flavonoid compound, luteolin can penetrate the blood-brain barrier and possesses anti-inflammatory properties. It has demonstrated efficacy in reducing inflammatory factors such as TNF-α in models of Alzheimer’s disease and autism spectrum disorder (97, 98). Notably, in a transgenic mouse model of Huntington’s disease (HD), luteolin treatment significantly extended lifespan by 25.5%, improved motor coordination and balance, reduced serum neurofilament light chain (NfL) levels (a marker of neuronal damage), and decreased mutant huntingtin protein aggregation in the cortex, hippocampus, and striatum (99). These findings suggest that luteolin holds promise as a therapeutic agent for Huntington’s disease, a condition for which no disease-modifying treatment is currently available. Additionally, blocking the IL-34/CSF1R signaling pathway with agents like PLX3397 can suppress pathological activation of microglia in the striatum, thereby mitigating neuronal damage (43, 100).

Given that a direct link between IKKβ and the IL-34/CSF1R cascade remains unproven, future work should focus on identifying the intermediate molecules that bridge these two signaling pathways. It is also essential to determine the downstream effectors by which IL-34/CSF1R-activated microglia aggravate the damage of medium spiny neurons. Furthermore, comparative studies can be conducted to uncover the differential regulatory effects of wild-type HTT and mutant HTT on IL-34 expression. These mechanistic explorations will deepen our understanding of the pathogenic roles of IL-34 in Huntington’s disease.

### 6.4 Prion diseases

Prion diseases, such as scrapie, are a group of infectious neurodegenerative disorders. Their pathology is characterized by the misfolding of cellular prion protein (PrPC) into pathological prion protein isoforms (PrPSc), which accumulates in the central nervous system, leading to neuron loss, along with proliferation and activation of astrocytes and microglia (101–103). Genetic ablation of global *Il34*, which results in microglial deficiency, leads to markedly increased deposition of proteinase K-resistant PrPSc and accelerated disease progression in mouse models. This finding provides compelling evidence that microglia exert a pivotal protective effect during prion pathogenesis (104). With regard to IL-34, prion infection does not elevate its expression but profoundly changes its cellular distribution. In normal mouse brain tissue, IL-34 is predominantly localized to neurons in the cortex, hippocampus, and thalamus. However, following prion infection, its cellular distribution in these brain regions shifts from neurons to activated astrocytes and microglia (105). Notably, the transcription and expression levels of CSF1R are significantly upregulated following infection, suggesting that the IL-34/CSF1R signaling axis may participate in prion disease-associated neuroinflammation through the alteration of IL-34’s cellular source. Therapeutic targeting of this pathway enables precise regulation of microglial function and proliferation. Such an approach may enhance the phagocytic and degradative capacity of microglia, thereby promoting the clearance of PrPSc. Importantly, complete microglial depletion and selective IL-34 intervention produce distinct outcomes and carry different adverse effects. Global elimination of microglia profoundly impairs innate immune surveillance and debris clearance in the central nervous system, robustly exacerbates PrPSc accumulation and prion disease progression, and also disrupts brain homeostasis, neuronal survival and blood–brain barrier integrity, bringing severe off-target risks. In contrast, specific IL-34 modulation only restrains excessive microglial proliferation rather than fully depleting this cell population, which largely preserves the basal protective functions of resident microglia. Nevertheless, IL-34 intervention may still interfere with physiological microglial maintenance and tissue immune balance, and could cause mild neuroinflammatory disturbances. Compared with pan-microglia ablation, targeting IL-34 achieves better tissue selectivity and fewer systemic side effects, representing a relatively safer strategy for prion disease treatment.

### 6.5 Ischemic stroke

Ischemic stroke is an acute cerebrovascular disease characterized by localized cerebral ischemia and hypoxia resulting from arterial occlusion, leading to neurological deficits (106, 107). This condition is commonly triggered by thrombi or emboli that interrupt cerebral blood flow, initiating a cascade of pathological responses including neuroinflammation, oxidative stress, and cell death (108, 109). Clinically, it presents as sudden neurological dysfunction, a process modulated by immune and inflammatory mediators (110, 111).

Studies have indicated that serum IL-34 levels in patients with acute ischemic stroke (AIS) are significantly higher than in healthy controls, and IL-34 has been confirmed as an independent predictor of AIS occurrence and functional prognosis (112). Additionally, in the prognosis of ischemic stroke, serum IL-34 levels are significantly elevated in patients with post-stroke cognitive impairment and are positively correlated with the degree of cognitive dysfunction (113). Although serum IL-34 has been identified as a valuable biomarker for acute ischemic stroke and post-stroke cognitive impairment, the function of endogenous IL-34 in the ischemic stroke is still unclear. Future work should therefore characterize CNS-resident IL-34, elucidate its roles in regulating local neuroinflammation, microglial activity and blood-brain barrier stability, and further explore the therapeutic potential of targeting the IL-34/CSF1R axis for ischemic stroke.

Experimental evidence demonstrates that IL-34 induces the proliferation of microglia expressing CSF1R, and promotes the differentiation of monocytes into a subset of macrophage with a CD14^high^ CD163^high^ CD80^low^CD86^low^ immunoregulatory phenotype through CSF1R signaling pathway (114). This process reduces the release of pro-inflammatory factors, protects the integrity of the blood-brain barrier (BBB), and alleviates brain damage after ischemia (78). IL-34 also facilitates monocyte differentiation into immunosuppressive macrophages and to protect blood-brain barrier integrity by upregulating tight junction proteins in endothelial cells. Collectively, these findings suggest that the CNS IL-34/CSF1R axis may be a promising therapeutic target for neuroinflammatory disorders including ischemic stroke (78, 114).

### 6.6 HIV-associated neurocognitive disorder

HIV-associated neurocognitive disorder (HAND) is a neurological complication of HIV infection characterized by progressive decline in memory, cognitive, motor, and behavioral functions (115, 116). The underlying pathology involves persistent HIV infection of brain macrophages and microglia (117, 118), which release inflammatory cytokines, triggering neuroinflammation that ultimately leads to neuronal injury and cognitive impairment (118, 119).

Studies have shown that in a simian immunodeficiency virus (SIV) encephalitis model, the two CSF1R ligands exhibit differential expression: CSF-1 is primarily expressed in meningeal tissues and perivascular accumulating macrophages, while IL-34 is more abundant in parenchymal cells, particularly neurons (120). Although co-localization of IL-34 with CD163 in the brain parenchyma was not significant, neuron-secreted IL-34 can act on myeloid cells, binding to CSF1R on their surface and increasing the expression of CD163 in primary monocytes. This suggests that IL-34 may promote and/or prolong a CSF1R-dependent, disease-context-dependent macrophage and microglial response in the brains of SIV-infected animals with encephalitis, with features consistent with an immunoregulatory and repair-associated state described in the context of HIV neuropathogenesis. This phenomenon has been validated *in vitro*: treatment of primary human and rhesus macaque peripheral blood mononuclear cells with IL-34 upregulates CD163 expression, and the addition of GW2580 (a highly specific receptor tyrosine kinase inhibitor for CSF1R) attenuates this effect. This indicates that the IL-34/CSF1R signaling axis is involved in maintaining the CD163+ phenotype (120). In addition, macrophages differentiated from human peripheral blood monocytes with IL-34 exhibit stronger resistance to HIV-1 infection compared to those induced by CSF-1, possibly due to the expression of higher levels of intrinsic restriction factor genes in IL-34-induced macrophages (44).

These findings indicate that IL-34 promotes the maintenance of CD163^+^ macrophages and microglia through the CSF1R signaling pathway and IL-34 dependent macrophages exhibit greater resistance to active HIV-1 infection, pointing to a critical role of IL-34 in regulating viral latency and central nervous system neuropathogenesis.

### 6.7 Multiple sclerosis

Multiple sclerosis is a chronic inflammatory disorder of the central nervous system with an autoimmune basis, pathologically characterized by immune‐mediated demyelination and concomitant neuronal degeneration (121). This pathogenesis involves abnormal T cell-driven autoimmune reactions, leading to inflammatory demyelination of the central nervous system, which ultimately causes impaired nerve conduction and progressive motor dysfunction (122, 123). Studies have shown that multiple sclerosis is associated with the alterations of microglia and interactions with various cytokines (124, 125). However, the specific pathological mechanisms still require further research.

Although no clear correlation with clinical or imaging activity has been established, IL-34 is considered a potential participant in MS pathogenesis, possibly serving a role analogous to the IgG index (126). Experimental evidence indicates that IL-34 levels in cerebrospinal fluid are significantly elevated in MS patients compared to controls with non-inflammatory neurological diseases. IL-34 promotes the formation of an autophagy-dependent microglial subset in cortex. Loss of this subset in aged mice exposed to autoimmune neuroinflammation leads to neuronal and glial cell death, increasing mortality; conversely, microglial expansion mediated by IL-34 exhibits a protective effect. Meanwhile, CSF-1 is also upregulated in multiple sclerosis models and sustains pathogenic myeloid cells, suggesting that CSF-1 may be a more critical therapeutic target for alleviating demyelination and gray matter pathology (37). In addition, vitamin D signaling can activate the expression of IL-34 in primary murine neurons and the N2a neuroblastoma cell line. IL-34 treatment of LPS-activated primary murine microglia and BV-2 microglia cells reduces pro-inflammatory cytokine release and enhances the expression of anti-inflammatory transcripts (127). However, the anti-inflammatory effects of vitamin D on microglia are only partially mediated by IL-34, as neutralizing IL-34 in vitamin D-treated neuronal conditioned media only reversed the suppression of IL-6, leaving other inflammatory markers unaffected (127), thereby alleviating central nervous system autoimmunity. Other studies have shown that IL-34 is involved in the immunosuppressive function of CD8^+^ regulatory T cells, which modulate immune responses by secreting factors such as interferon-γ, TGF-β, and IL-34. In patients with multiple sclerosis, dysfunction of these cells is closely associated with disease severity during relapses (128).

Overall, potential therapeutic strategies for multiple sclerosis include supplementing vitamin D, which induces neuronal release of IL-34 along with other immunomodulatory molecules to promote microglial secreting anti-inflammatory cytokines and neuroprotection; as well as restoring CD8^+^ regulatory T cell function to enhance IL-34 secretion (128).

### 6.8 Neuropsychiatric disorders

Neuropsychiatric disorders are a complex category of diseases that significantly impact cognition, emotional regulation, behavior, and mental health. They primarily include Alzheimer’s disease (AD), anxiety disorders, epilepsy, schizophrenia, autism spectrum disorder (ASD) (129) and Kleine-Levin syndrome (130). The occurrence and progression of these diseases are related to the interaction of genetic, molecular, and environmental factors (131). Recent studies have highlighted the crucial role of immune regulation and neuroinflammation in pathogenesis (131, 132), Imaging scans further revealed the increased translocator protein (TSPO)+ microglia in the brains of patients with various neuropsychiatric disorders (133), providing strong evidence.

Notably, IL-34 levels are significantly decreased in the serum of patients with obsessive–compulsive disorder (OCD) compared to healthy controls and are negatively correlated with disease severity (134). In contrast, in the cerebrospinal fluid of patients with Kleine-Levin syndrome, IL-34 levels are elevated, accompanied by microglial changes and disruption of the blood-brain barrier permeability (135), suggesting that IL-34 participates in disease processes through distinct immunoregulatory mechanisms across neuropsychiatric disorders. In addition, IL-34 has been shown to drive the differentiation of human peripheral blood monocytes into induced microglia-like cells (iMGs) within 14 days. These iMGs exhibit functional characteristics such as phagocytic activity and cytokine secretion, holding potential for clinical applications (136). This method establishes a new platform for drug screening and personalized treatment (137), which is practical in the research of many brain diseases (138).

## 7 Discussion

In the CNS, IL-34 critically orchestrates the tripartite communication among neurons, microglia, and the developing brain, with significant implications for both neurodevelopmental processes and disease pathogenesis. During critical postnatal windows, IL-34 expression peaks in excitatory neurons, providing a spatially and temporally restricted signal that calibrates microglial activity. This neuron-derived IL-34 is not merely a survival factor for microglia but acts as a functional modulator, restraining excessive synaptic pruning and thereby preserving circuit integrity (20). Conversely, the downregulation of IL-34 in niches of active neurogenesis, such as the hippocampus, creates a permissive microenvironment that dampens microglial surveillance, facilitating the integration of newborn neurons (76). This dynamic, bidirectional crosstalk underscores IL-34 as a key mediator of neurodevelopmental plasticity, ensuring that microglial functions—from synaptic refinement to trophic support—are precisely tuned to the needs of the maturing brain.

**Figure 3:**
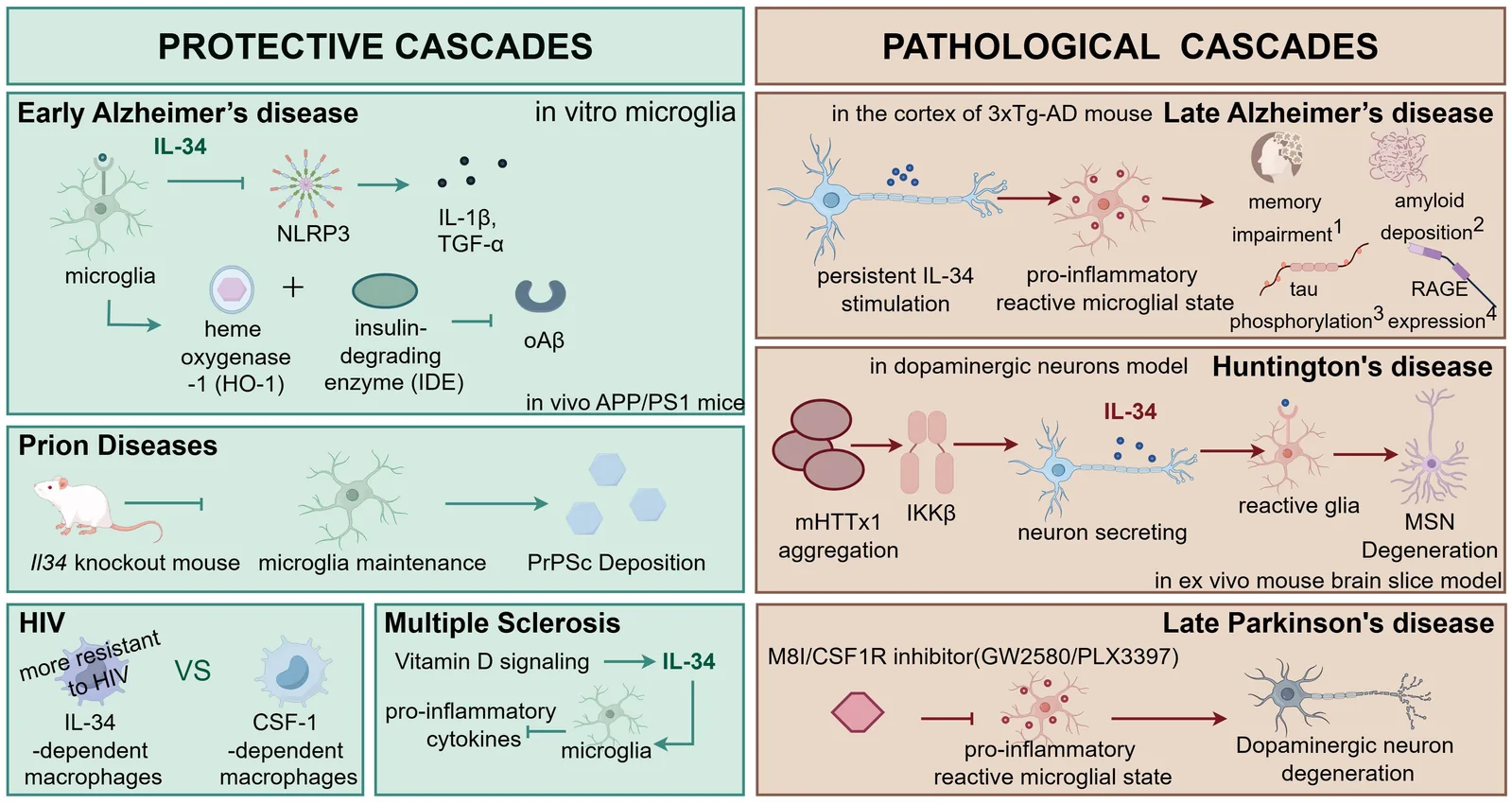
Schematic representation of the protective versus pathological cascades mediated by IL-34/CSF1R signaling in neurodegenerative pathologies. Interleukin-34 (IL-34) plays opposing, context-dependent roles across neurodegenerative conditions, classified into early protective/reparative pathways (left, green panel) and late-stage pathological/pro-inflammatory pathways (right, orange panel). Protective Cascades (Left Panel): Early Alzheimer’s Disease (AD): Secreted IL-34 acts on homeostatic microglia to inhibit NLRP3 inflammasome activation, suppressing the release of pro-inflammatory cytokines such as IL-1β and TNF-α. Simultaneously, IL-34 upregulates microglial expression of heme oxygenase-1 (HO-1) and insulin-degrading enzyme (IDE), facilitating the clearance of neurotoxic oligomeric amyloid-β (oAβ). Prion Diseases: In Il34 knockout mouse model, global deletion of Il34 leads to severe microglial depletion, resulting in increased accumulation of proteinase K-resistant scrapie prion protein (PrPSc) deposits and accelerated disease progression, demonstrating the essential protective role of IL-34-dependent microglia in physiological surveillance. Human Immunodeficiency Virus (HIV): *In vitro*, human monocyte-derived macrophages differentiated via IL-34 show enhanced resistance to HIV-1 infection compared to CSF-1-derived macrophages, attributed to elevated baseline expression of host restriction factors. Multiple Sclerosis (MS): Vitamin D signaling enhances IL-34 production, which acts on activated microglia to attenuate pro-inflammatory cytokine secretion and restore immune homeostasis. Pathological Cascades (Right Panel): Late Alzheimer’s Disease (AD): In the cortex of late-stage AD models (e.g., 3xTg AD mice), persistent and excessive IL-34 stimulation drives microglia into a chronic pro-inflammatory reactive state, accelerating amyloid deposition, tau hyperphosphorylation, and cognitive/memory impairment. Huntington’s Disease (HD) (Proposed/Unconfirmed Pathway): Aggregation of mutant huntingtin exon 1 (mHTTx1) activates IKKβ, which is hypothesized to induce neuronal secretion of IL-34. Paracrine IL-34 stimulates microglia toward a reactive phenotype, thereby promoting the degeneration of striatal medium spiny neurons (MSNs) in brain slice models. Note: The direct regulatory link between IKKβ and the IL-34/CSF1R axis remains to be fully confirmed. Late Parkinson’s Disease (PD): In models of dopaminergic neuron degeneration, pharmacological blockade using matrix metalloproteinase-8 inhibitors (M8I) or selective CSF1R inhibitors (GW2580, PLX3397) suppresses microglial hyperactivation and halts neuronal degeneration. Created with Figdraw.

Dysregulation of the IL-34-microglia-neuron axis can induce microglial polarization towards either pro-inflammatory or anti-inflammatory phenotypes, playing protective or detrimental roles at different stages of neurological diseases (**Figure 3**). Regarding the anti-inflammatory/protective phenotype, during neurodevelopment, appropriate IL-34 expression supports normal microglial synaptic pruning, whereas its deficiency leads to aberrant microglial phagocytosis of synapses, resulting in disrupted neural connectivity and behavioral phenotypes such as cognitive impairment and anxiety-like behaviors (20). In the early stages of neurodegenerative diseases like Alzheimer’s disease, neuron-derived IL-34 may help maintain microglial clearance function and anti-inflammatory responses, supporting the elimination of toxic protein aggregates, while the loss of IL-34 compromises these protective mechanisms (22). However, in the chronic or late stages of the aforementioned neurodegenerative diseases, IL-34 signaling may become dysregulated in late-stage disease, contributing to a context-dependent microglial state with inflammatory and neurodegenerative features and thereby exacerbating neuronal injury (23). Similarly, in Huntington’s disease, neuronal IL-34 overexpression induced by mutant huntingtin activates microglia via CSF1R, thereby aggravating striatal neurodegeneration (43). Thus, the function of the IL-34-microglia-neuron axis is highly context-dependent, and its dynamic balance between anti-inflammatory repair and pro-inflammatory injury determines its pathological trajectory in neurological disorders.

Given that the IL-34-microglia axis can exert either protective or detrimental roles at different stages of neurological diseases, modulating or replacing dysfunctional microglia has emerged as a promising therapeutic strategy. In recent years, microglial depletion therapy has attracted considerable attention. This strategy utilizes CSF1R inhibitors to eliminate endogenous microglia, allowing for cell repopulation upon treatment cessation or achieving myeloid cell reconstitution through transplantation, aiming to “reset” the dysfunctional state of microglia (139, 140). In animal models of diseases such as AD and HD, this strategy has demonstrated preliminary efficacy in reducing pathological protein aggregation and improving mouse behavior (141, 142). A recent study further revealed that in Alzheimer’s disease models, microglial depletion effectively reduces APOE4-associated Aβ and tau pathologies (APOE4 > APOE3), suggesting that microglial depletion may be a viable approach to counteract the high-risk effects of APOE4.

However, microglial depletion therapy still faces numerous challenges. First, the efficacy is highly dependent on disease models and therapeutic time windows. A systematic review and meta-analysis revealed that short-term microglial depletion in Alzheimer’s disease models did not significantly reduce overall Aβ levels, and longer depletion periods were required to observe positive effects on Aβ pathology. In Parkinson’s disease models, some studies reported exacerbation of motor dysfunction. Given that improving motor symptoms is the primary clinical goal for treating Parkinson’s disease, caution is warranted when targeting microglia as a therapeutic strategy (143). Moreover, microglia are not only involved in pathological inflammation, but also participate in complement-dependent synaptic pruning, development, and memory-related homeostatic processes. Thus, extensive depletion disrupts physiological synaptic elimination, thereby affecting normal memory forgetting or network remodeling. Furthermore, as discussed earlier, microglia can exert both protective and detrimental dual roles at different disease stages, making simple depletion less rational than “precise modulation.” In this context, emerging microglial replacement strategies may offer a more refined alternative—selectively eliminating dysfunctional microglia and reconstituting central nervous system homeostasis through newly generated cells (144). Therefore, future research should focus on developing more cell-specific and stage-adaptive regulatory strategies based on a clear understanding of the dynamic changes in IL-34 signaling.

Compared with direct microglial depletion, IL-34-targeted therapy presents distinct advantages and differences. IL-34 intervention precisely regulates microglial activity rather than ablating the entire cell population. It circumvents the unstable efficacy of microglial depletion across different disease models and time windows, and protects microglia-mediated physiological processes including synaptic pruning and memory homeostasis. Benefiting from the dynamic characteristics of the IL-34-microglia axis, this strategy can be adjusted to match different disease stages. In contrast, universal microglial depletion is a non-selective approach: it relieves pathological protein deposition in some neurodegenerative models but may worsen clinical symptoms and disrupt normal brain function. Considering these discrepancies, IL-34-based modulation is a more reasonable and safer option. Therefore, future research should focus on developing more cell-specific and stage-adaptive regulatory strategies based on a clear understanding of the dynamic changes in IL-34 signaling.

While strategies targeting parenchymal microglia aim to reset CNS homeostasis, the meningeal immune compartment—situated at the interface between the periphery and the brain—has recently emerged as another key modulator of neuropathological processes. Given IL-34’s role in both inflammatory neurological disorders and neural homeostasis, the regulatory machinery of meningeal immunity presents a promising therapeutic target. Therefore, a deeper understanding of its immunogenic mechanisms is crucial. Research has shown that neurons can communicate with macrophages in the meninges by releasing peptides, and this neuroimmune axis may suppress host defense against pathogens, thereby exacerbating meningitis (145), suggesting meningeal immunity profoundly influences CNS homeostasis. Furthermore, meningeal immunity participates in post-injury CNS recovery, chronic neurodegenerative diseases, and even higher brain functions (146). Therapeutically, the meninges (particularly the dura mater) are more accessible for peripheral entry than the CNS parenchyma.

As key mediators of meningeal immunity, meningeal macrophages play an essential role, making it imperative to understand their developmental and homeostatic regulation. However, most studies on IL-34-mediated macrophage differentiation have focused on microglia, and the specific mechanisms of its action in meningeal macrophages have only recently been elucidated. However, the precise mechanism by which IL-34 controls the early developmental colonization and functional differentiation of meningeal macrophages remains unresolved. For example, during embryonic development, how do meningeal macrophage precursors migrate to specific meningeal locations (such as the dura mater or leptomeninges) and establish residency? Does IL-34 act as a direct driver or does it shape the intercellular microenvironment to drive migration? Which specific cell types primarily secrete IL-34 into the meningeal spaces during early development? The resolution of these questions is critical for understanding the organizational logic of the meningeal immune system.

While IL-34/CSF1R axis acts as a central regulatory module governing CNS cellular differentiation and neuroimmune homeostasis, a key translational gap remains: the vast majority of current mechanistic evidence derives from rodent or zebrafish models, whereas human evidence is predominantly observational or biomarker-based. Consequently, clinical claims regarding IL-34-targeted therapies must be interpreted with caution until validated in clinical settings. Furthermore, several fundamental open questions remain, including those mentioned above. Future studies leveraging multi-omics approaches, intravital imaging, and human brain organoids will be essential to bridge this translational divide, unravel spatiotemporal regulatory networks, and ultimately pave the way for precise neuroimmune interventions.
